# Xanthogranulomatous Pyelonephritis and Its Differential Diagnoses: An In-Depth Case Review

**DOI:** 10.7759/cureus.19133

**Published:** 2021-10-29

**Authors:** María Herrera-Bedoya, Camilo A Avendaño-Capriles, Elias Zakzuk-Martinez, Jesus Barrera

**Affiliations:** 1 Pathology, Ciencias de la Salud, Fundación Universidad del Norte, Barranquilla, COL; 2 Foundations of Clinical Research (FCR) Program Scholarship, Harvard Medical School, Boston, USA; 3 Department of Medicine, Universidad del Norte, Barranquilla, COL; 4 Research Group on Hospital Management and Health Policies, Universidad de la Costa, Barranquilla, COL; 5 Epidemiology and Public Health, Ciencias de la Salud, Fundación Universidad del Norte, Barranquilla, COL

**Keywords:** renal cell carcinoma, renal neoplasms, renal tuberculosis, malakoplakia, xanthogranulomatous pyelonephritis

## Abstract

Xanthogranulomatous pyelonephritis is a rare chronic infectious process of the kidney, which has been described in three different forms: diffuse, segmental, and focal. It is also known as the great simulator since its clinical, radiological, and histopathological manifestations tend to be confused with other entities. We describe a case of a 55-year-old male patient with two months of clinical manifestations characterized by a 7x7-cm palpable mass in his right lumbar region, which was hot and painful upon touch and increasing in size. This article aims to present a case of xanthogranulomatous pyelonephritis and compare it with its primary differential diagnoses. It is evident that despite the condition being considered a simulating pathology, some key differences can be found to identify and distinguish it.

## Introduction

Xanthogranulomatous pyelonephritis is an uncommon subtype of pyelonephritis, representing 0.6% of all varieties of its chronic presentation. Some of its typical signs and symptoms include fever, loss of weight, and flank pain, but its presentation is mainly variable. Despite that, it might have an insidious course, and it is vital to keep neoplasms in mind for its differential diagnosis. Therefore, the diagnostic confirmation of this disease can be challenging [1].

Currently, we rely on significant advances in renal pathology that have resulted in quick methods with great sensibility and specificity and specific clinical characteristics that enable physicians to establish an early diagnosis. As a result, we find ourselves confronted with increasingly prevalent illnesses of rising incidence whose physiopathology, treatment, and survival rate can be clearly estimated. Still, not much is known about pathologies that simulate typical renal lesions, or they remain underdiagnosed due to a lack of knowledge about their existence or detection methods. Furthermore, this issue varies from place to place due to sociodemographic factors, which enhance diagnostic possibilities. In light of this, this report presents the case of a patient with xanthogranulomatous pyelonephritis and compares its main findings with those of different renal tumors and pseudotumors that can imitate common renal and genitourinary pathologies, which have vastly different etiologies.

## Case presentation

A 55-year-old male patient from Soledad, Colombia, presented to the primary care center due to a two-month clinical condition characterized by a 7x7-cm palpable mass in his right lumbar region, which was hot and painful upon touch and progressively increasing in size. Additionally, the patient stated that he occasionally experienced fever and urinary symptoms and had lost approximately 8.4% (4 kg) of his body weight (48 kg). On admission, he weighed 44 kg, was afebrile, and without urinary symptoms. The rest of the physical examination was normal, except for the palpable mass previously described and right costovertebral angle tenderness. The patient's past medical history included arterial hypertension, a left ventricular ejection fraction (LVEF) of 49%, and the removal of a giant auricular myxoma along with mitral valve vegetation abscission. In addition, the patient brought a urinary tract echography performed five days before the admission, which showed a 5x1.2-cm mobile anechoic structure within the right lumbar region.

**Figure 1:**
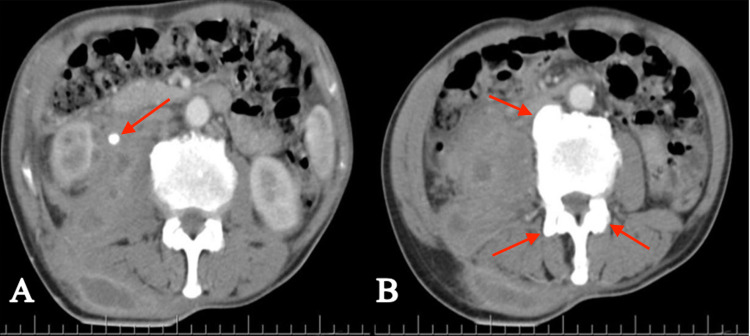
Abdominal CT scan with contrast A) Right kidney with markedly increased size; the presence of a 7.5-mm and 409-UH lith is apparent in the proximal third of the right ureter. Adjacent to it, an image compatible with a collection of heterogeneous aspects is evident, which compromises the right paracolic gutter and muscular planes (right oblique muscle, psoas muscle, and paravertebral muscles). The collection measures approximately 17.7x9.8x7.9 cm and has a volume of 712 cc. B) Degenerative changes at the level of the dorsolumbar area, indicated by the presence of osteophytes CT: computed tomography

Initial laboratory results reported normocytic and hypochromic anemia, thrombocytosis, and leukocytosis with slight neutrophilia. Urinalysis and urinary gram stain were positive, which prompted the initiation of empirical therapy with broad-spectrum antimicrobials. Subsequently, the urinary culture revealed the presence of AmpC Escherichia coli (E. coli). Given these results, the medication had to be changed to ertapenem to extend the antimicrobial spectrum. An abdominal CT scan with contrast was performed, which revealed a calculus located in the proximal third of the right ureter. It had a diameter of 7.9 mm and a density of 409 UH. Furthermore, a 712-cc collection was evident, affecting the ipsilateral paracolic gutters and iliac psoas muscle, and extending towards cellular tissues and oblique and paravertebral muscle planes (Figure 1).

Due to the clinical and radiological findings, and to achieve optimal conditions, an exploratory lumbotomy was performed, and drainage of the retroperitoneal collection was conducted. During the procedure, caseous material was discovered in the right iliac fossa and paravertebral spaces. The kidney firmly adhered to the paracolic gutter and its adjacent muscular planes. The area was cleaned, and an 18 French Nelaton catheter was placed. Renal tuberculosis (TB) was suspected. Hence, the caseous material was sent for a microbiological analysis, which yielded a negative result for TB.

**Figure 2:**
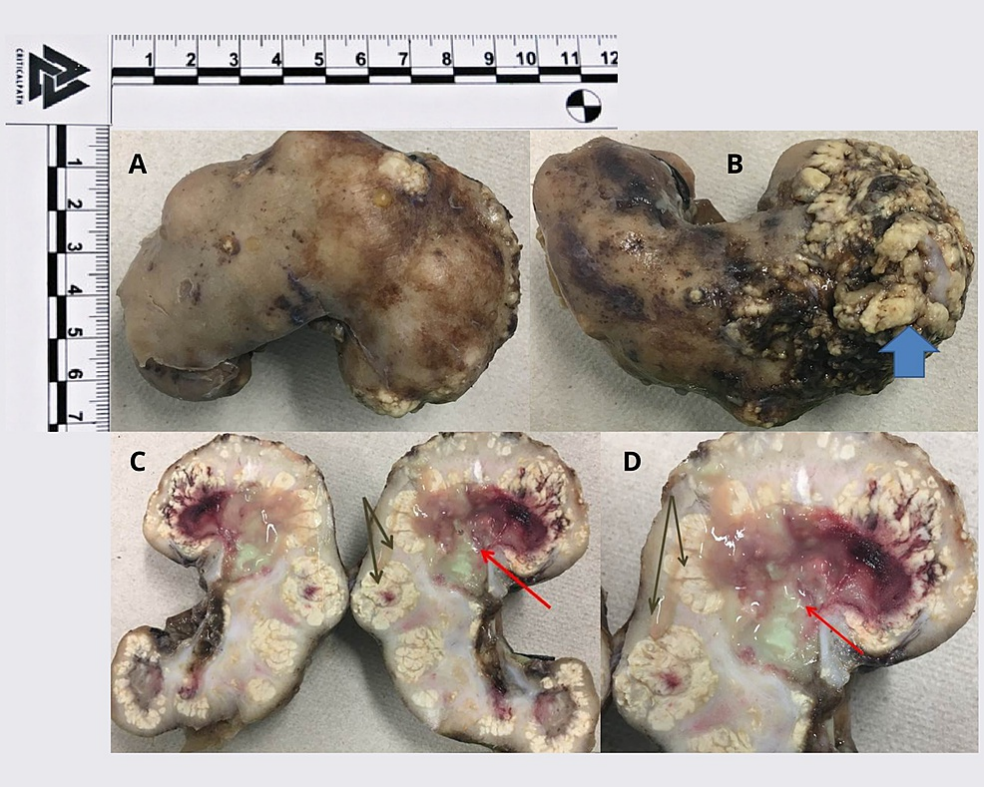
Pathological study A) Nephrectomy product, 125 g – measuring 9x5x3.5 cm. B) Blue arrow: bloodstained zone on the posterior face, superior pole – measuring 4.5x4.5 cm. C-D) Black arrow: loss of corticomedullary differentiation, yellow irregular, and nodular areas. Red arrow: cavitated areas containing greyish yellow and hemorrhagic material

**Figure 3:**
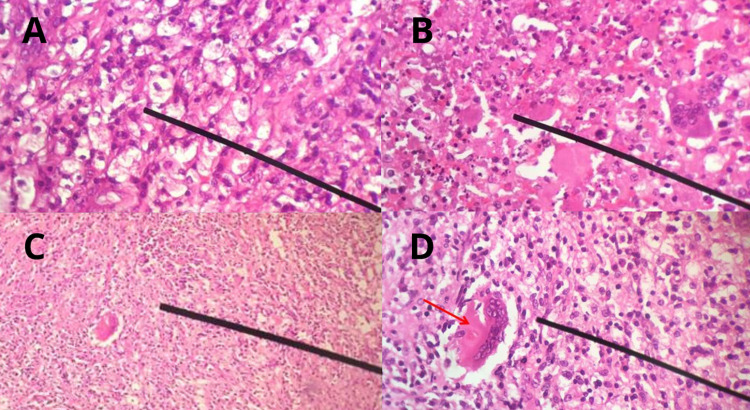
Histopathological study A) Predominantly histiocytic inflammatory infiltrate – 40X. B) Inflammatory infiltrate with histiocytes and polymorphonuclear neutrophils – 40X. C) Histiocytic inflammatory infiltrate – 4X. D) Histiocytic inflammatory infiltrate – red arrow: giant multinucleated cell – 40X

The patient was monitored, and no deterioration of his clinical state was observed. A control abdominal CT scan showed a diminished retroperitoneal collection with the involvement of cellular tissues and paravertebral and muscular planes. Based on these findings, a decision was made to finish the antimicrobial treatment and perform a radical right nephrectomy with the corresponding histopathological analysis of the removed material. The report described renal parenchyma with areas of fibrosis, sclerosis, and a lymphohistiocytic inflammatory infiltrate. Plasmocytes, foamy histiocyte accumulation, giant multinucleated cells, and an inflammatory infiltrate of polymorphonuclear neutrophils forming microabscess were evident as well (Figures 2, 3). These findings confirmed the diagnosis of xanthogranulomatous pyelonephritis.

**Figure 4:**
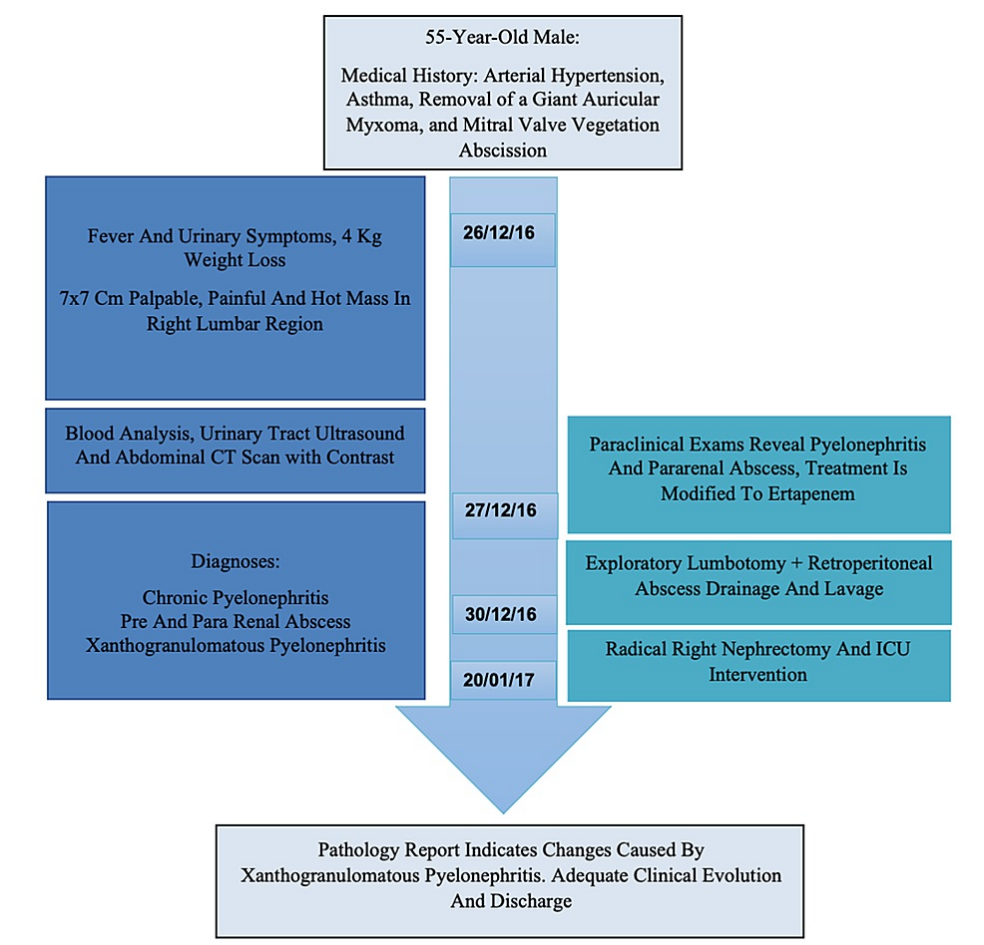
Timeline with significant dates CT: computed tomography; ICU: intensive care unit

The patient was then shifted to the intensive care unit for postoperative vigilance, which led to a favorable clinical course without any further complications. Afterward, he was transferred to the hospitalization area and was discharged from the institution when he fully recovered from the procedure. On release, the patient received a hospital discharge summary with recommendations and a set of follow-up appointments (Figure 4).

## Discussion

Xanthogranulomatous pyelonephritis is a variant of chronic pyelonephritis in the setting of chronic obstruction due to renal calculi, which may lead to massive unilateral destruction of the kidney with the presence of granulomatous tissue that contains lipid-filled macrophages. Three forms of this condition have been described: diffuse, segmental, and focal. The diffuse type is the most common one, and there are three stages associated with it [2]. The first one is the nephric stage, which is confined to the renal parenchyma; stage II, or the perinephric stage, involves the perirenal space and Gerota's fascia; and stage III, or the paranephric stages, refers to the involvement of the pararenal area and retroperitoneal structures [3].

This disease could occur due to defective phagocytosis of bacteria by macrophages during an infection caused by one of the microorganisms most commonly associated with urinary tract infections, such as E. coli, like in this case, Proteus mirabilis, Pseudomonas aeruginosa, Enterococcus faecalis, Staphylococcus aureus, and Klebsiella pneumoniae [4]. Its clinical presentation is nonspecific and can simulate several pathologies, and hence it is frequently confused with some of them, e.g., renal carcinoma: since both diseases have a similar clinical presentation and radiographic appearance [5]. Renal abscesses are an essential part of its differential diagnosis as well, along with renal and urothelial neoplasms [6,7].

Its diagnosis is histopathological. Therefore, it must be histologically differentiated from two similar inflammatory conditions: renal parenchymal malakoplakia and megalocytic interstitial nephritis [8]. In the case of our patient, renal TB was excluded from the possible diagnoses because of his demographic characteristics, personal background, clinico-radiological findings, and natural history of the disease. All of this led to the analysis of the caseous material, which yielded a negative result.

Due to the nonspecific symptoms and shifting clinical presentation found in patients with xanthogranulomatous pyelonephritis [9], the amount of possible similar pathologies for differential diagnosis is even more tremendous. This forces physicians to use the clinical manifestations of a particular patient to try to ascertain the most likely diagnosis (Table 1).

**Table 1:** Differential diagnoses in patients with renal masses based on clinico-radiological characteristics* *[1-13] +: frequent in the disease; -: not frequent in disease; ±: may or may not be present in the disease

Symptoms	Differential diagnoses				
	Renal TB	Urothelial carcinoma	Renal cancer	Xanthogranulomatous pyelonephritis	Malakoplakia
Fever	±	±	±	+	+
Hematuria	+	+	+	+	+
Leukocytosis	-	-	-	+	±
Weight loss	±	±	+	+	-
Anemia	-	±	+	±	-
Pain	±	+	+	+	+
Macroscopic appearance	Yellowish-white lesions	Urinary tract infiltrating mass	Depending on the type of cancer	Yellowish-necrotic	Yellowish infiltrate
Imaging	Parenchymatous scars and calcifications	Solid mass in the urinary tract	Solid renal mass	A heterogenous unilateral mass associated with renal calculus	Cystic or solid mass, usually unilateral
Invasion into neighboring structures	+	+	±	±	±

All the previously mentioned diseases constitute a list of possible pathologies to consider in the differential diagnosis in patients who present a renal mass and fit into the sociodemographic context of these diseases. However, regarding granulomatous conditions, sometimes a proper diagnosis can only be performed with the aid of histopathological examination [10] due to several clinical and radiological similarities among various pathologies, which do not allow physicians to precisely distinguish between the diagnostic possibilities. Malakoplakia is a case in point, which shares several clinical symptoms with xanthogranulomatous pyelonephritis, like fever, flank pain, features of urinary tract infections, or even signs of perinephric abscess [14].

The main difference is that xanthogranulomatous pyelonephritis is usually associated with the presence of renal calculi. In contrast, in malakoplakia, their sighting is not mandatory for a diagnosis, nor are they required to develop the disease. Xanthogranulomatous pyelonephritis is associated with renal calculi in approximately 80% of the cases, according to some studies [8,9]. Consequently, the only proper way both pathologies could be differentiated would be through a histopathological analysis where malakoplakia presents von Hansemann cells (eosinophilic histiocytes) and Michaelis-Gutmann bodies (intracytoplasmic basophilic inclusions). In contrast, xanthogranulomatous pyelonephritis only shows lipid-filled macrophages (foamy histiocytes) [15,16].

Given that the loss of parenchyma is progressive and eventually leads to renal failure, the treatment of choice for the diffuse type is nephrectomy, with resection of all the surrounding tissues affected. After removal, the prognosis is usually good [2,17]. For the focal type, antibiotic therapy alone may be an option. Still, before initiating the treatment, a biopsy must be performed to confirm the diagnosis and exclude cancer [5,18]. However, for the focal or segmental types, partial nephrectomy may be an option as well, especially in patients with a solitary kidney [19].

It is essential to mention that this disease is usually unilateral and more common in females. Almost always, it affects the right kidney [2]. In the case described, our patient was a male, but the other two factors were present. Furthermore, he had many of the findings related to the disease, such as fever, urinary symptoms, weight loss, pain, leukocytosis, and anemia. However, as already mentioned, these are nonspecific and common in other diseases as well. Subtle details, such as the fact that malakoplakia and renal TB do not usually present with weight loss, nor anemia, that renal cancer rarely has leukocytosis, and that urothelial carcinoma usually does not have accompanying leukocytosis, nor weight loss, made us put xanthogranulomatous pyelonephritis at the top of our list for differential diagnosis.

Since the patient was from Latin America, along with the finding of caseous material within the abscess, the suspicion of renal TB was also deemed warranted, which was subsequently excluded from the differentials after performing the corresponding microbiological analysis. The presence of AmpC E. coli and the renal calculus reinforced the perception that our central differential had to be considered. That said, the abscess formation was probably due to the presence of xanthogranulomatous pyelonephritis, which is one of its possible complications. Others include the development of emphysematous pyelonephritis, perinephric abscess, psoas muscle abscess, nephrocutaneous fistula, nephrocolonic fistula, and ischemic colitis [20].

After the antimicrobial therapy was performed, we noticed that the abscess containing caseous material was reduced in size. However, we decided to perform a radical nephrectomy to achieve a definitive resolution. This choice was made since our patient had involvement of the retroperitoneal structures and pararenal space, which was suggestive of stage III diffuse xanthogranulomatous pyelonephritis and demanded an aggressive approach. Moreover, since the disease is progressive, the patient probably already had a nonfunctioning kidney due to the presence of extensive damage, which is mainly associated with advanced stages of the disease.

When the histopathological analysis was completed, the diagnosis was confirmed. This highlights the importance of keeping this disease in mind along with other pathologies mentioned in patients presenting with renal masses.

## Conclusions

An early diagnosis is of paramount importance in cases of xanthogranulomatous pyelonephritis to avoid further complications, such as the development of abscesses, involvement of surrounding structures, and progressive loss of renal parenchyma, which could result in a nonfunctioning kidney. Therefore, it is critical to always consider the clinical, imagenological, and histopathological differences discussed, which could help identify the presence of any of these uncommon diseases with more ease and employ a better diagnostic and therapeutic approach.
